# Alterations of 5-Hydroxymethylcytosine in Human Cancers

**DOI:** 10.3390/cancers5030786

**Published:** 2013-06-25

**Authors:** Christopher J. Mariani, Jozef Madzo, Erika L. Moen, Ali Yesilkanal, Lucy A. Godley

**Affiliations:** 1 Section of Hematology/Oncology, Department of Medicine, The University of Chicago, 5841 S. Maryland Ave., MC 2115, Chicago, IL 60637, USA; E-Mails: cmariani@uchicago.edu (C.J.M.); jmadzo@uchicago.edu (J.M.); emoen@uchicago.edu (E.L.M.); aeyesilkanal@uchicago.edu (A.Y.); 2 Committee on Molecular Pathogenesis and Molecular Medicine, The University of Chicago, Chicago, IL 60637, USA; 3 Committee on Cancer Biology, The University of Chicago, Chicago, IL 60637, USA

**Keywords:** 5-hydroxymethylcytosine, 5-methylcytosine, cancer, DNA methylation, epigenetics, ten-eleven translocation, TET

## Abstract

Prior to 2009, 5-methylcytosine (5-mC) was thought to be the only biologically significant cytosine modification in mammalian DNA. With the discovery of the TET enzymes, which convert 5-methylcytosine (5-mC) to 5-hydroxymethylcytosine (5-hmC), however, intense interest has emerged in determining the biological function of 5-hmC. Here, we review the techniques used to study 5-hmC and evidence that alterations to 5-hmC physiology play a functional role in the molecular pathogenesis of human cancers.

## 1. Introduction

### 1.1. The Cytosine Modification Pathway

In mammals, methylation of cytosine residues within DNA is catalyzed by a group of DNA methyltransferases (DNMTs): DNMT1, DNMT3A, and DNMT3B. DNMT1 is classified as a maintenance methyltransferase because it has high affinity for hemimethylated DNA and ensures that the daughter strand of DNA inherits the same methylation mark during DNA replication. For this reason, DNA methylation is said to be heritable. In contrast to DNMT1, DNMT3A and DNMT3B are classified as *de novo* methylases, although they also play some role in maintaining DNA methylation patterns as cells replicate [1]. Although 5-hydroxymethylcytosine (5-hmC) was identified in mammalian DNA in 1972 [2], it was not thought to have an important function and attention focused on 5-methylcytosine (5-mC). Accumulation of 5-mC at gene promoters is associated with gene repression [1]. 5-mC elicits this effect by binding various methyl binding proteins that then recruit histone deacetylases and other chromatin remodeling enzymes and corepressors [3].

**Figure 1:**
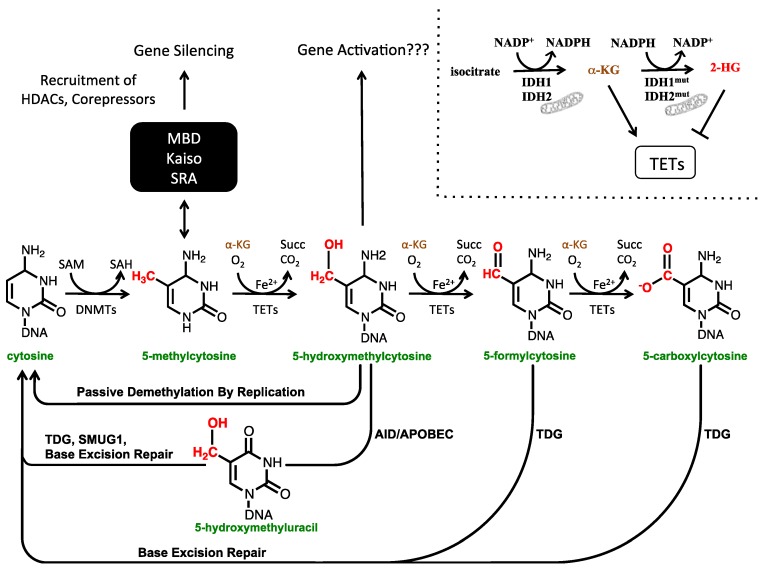
The cytosine modification pathway. DNMT1, DNMT3A, and DNMT3B methylate cytosine to form 5-methylcytosine (5-mC) using S-adenosyl methionine (SAM) as a methyl donor. 5-methylcytosine can then be oxidized to 5-hydroxymethylcytosine (5-hmC), 5-formylcytosine (5-fC), and 5-carboxylcytosine (5-caC) by the TET dioxygenases, which use α-ketoglutarate (α-KG), molecular oxygen, and iron as cofactors. Isocitrate dehydrogenase (IDH) enzymes produce α-KG by oxidation of isocitrate. IDH2 is found in the mitochondria. Mutated IDH proteins are capable of generating 2-hydroxyglutarate (2-HG), which inhibits the TET dioxygenases (top right). 5-mC at gene promoters represses gene transcription by binding methyl binding domain (MBD) proteins (MeCP2, MBD1, MBD2, MBD3, MBD4), Kaiso family proteins (Kaiso, ZBTB4, ZBTB38), and SRA domain proteins (UHRF1, UHRF2) that then recruit histone deacetylases (HDACs) and corepressors. 5-hmC likely has a role in activating gene transcription. Numerous demethylation pathways involving oxidized cytosine species have been proposed. 5-hmC may be an intermediate in passive demethylation by replication since it is not recognized by DNMT1. 5-hmC can also be deaminated by AID/APOBEC to give 5-hydroxymethyluracil which can than be excised and replaced with cytosine. Finally, thymine DNA glycosylase (TDG) can also directly remove 5-fC and 5-caC, which when repaired with base excision repair machinery yield an unmodified cytosine.

The ten-eleven-translocation 1 (TET1) enzyme was identified first as an MLL fusion partner in leukemia [4,5], but its biological function was not known until 2009, when it was identified as a dioxygenase capable of converting 5-mC to 5-hmC [6]. This discovery, combined with the observation that 5-hmC is present in embryonic stem cells (ESCs) [6] and the brain [7], ignited intense interest in 5-hmC. The TET enzymes oxidize 5-mC using Fe(II), molecular oxygen, and α-ketoglutarate [6]. α-ketoglutarate is generated by oxidation of isocitrate to succinate by the isocitrate dehydrogenase (IDH) enzymes IDH1 and IDH2, the latter of which is located in the mitochondria. Although initial work on the TET enzymes focused on their capacity to convert 5-mC to 5-hmC, it was later discovered that they can further oxidize 5-hmC to 5-formylcytosine (5-fC) and 5-carboxylcytosine (5-caC) (Figure 1) [8,9,10]. Although multiple cytosine modifications beyond 5-mC have now been identified, they differ in their abundance within the genome, with 5-hmC being present at a frequency around 100-fold higher than that of 5-fC and 5-caC [8]. This review will focus on 5-hmC, since it is the most abundant and well-studied of these novel cytosine species.

### 1.2. The Biological Functions of 5-hmC

As described above, 5-mC represses transcription at promoters by recruiting methyl-binding proteins, which then interact with other proteins to repress DNA transcription. At the least, conversion of 5-mC to 5-hmC may serve to release methyl-binding proteins from DNA, creating a chromatin state more facilitative towards transcription.

Oxidation of 5-mC to 5-hmC by the TET enzymes may also play a role in cytosine demethylation. Various mechanisms of demethylation involving the TET proteins have been proposed. The simplest of these demethylation mechanisms is a passive one, whereby hydroxymethylated cytosines are not recognized by DNMT1 during replication. As a result, a hydroxymethylated cytosine in the parent strand of DNA is replicated initially in the daughter strand as an unmodified cytosine (Figure 1). This passive mechanism is supported by the finding that DNMT1 has ~10-fold lower activity at hemi-hydroxymethylated CpGs compared with hemi-methylated CpGs [11]. Active, replication-independent methods of demethylation have also been proposed through various pathways. Prior to the discovery of 5-hmC, 5-fC, and 5-caC, one model of DNA demethylation involved deamination of 5-mC to thymine by an AID/APOBEC enzyme. In this pathway, the resulting thymine residue is excised by thymine DNA glycosylase (TDG) and base excision repair (BER) replaces the abasic site with cytosine [12,13]. This pathway can also operate on 5-hmC: AID/APOBEC can deaminate 5-hmC to 5-hydroxymethyluracil, which can then be excised by both TDG and single-strand selective monofunctional uracil-DNA glycosylase 1 (SMUG1) to give an abasic site that is repaired by BER [13,14]. Nonetheless, this pathway has been called into question as AID/APOBEC deaminases have been found to have limited activity at 5-mC, and even less at 5-hmC, due to the increasing steric bulk at the 5-position of these nucleotides [15,16]. Finally, TDG can also excise 5-caC and 5-fC directly, providing for an additional demethylation mechanism (Figure 1) [9,17]. Whether or not deformylases or decarboxylases exist that can convert 5-fC and 5-caC directly to cytosine is an open question [18,19,20,21].

Multiple publications have identified 5-hmC binding proteins, such as MBD3/NURD [22], UHRF1 [23], and MeCP2 [24]. Most recently, Sprujit *et al*. used quantitative mass-spectrometry to identify 5-hmC binding proteins in mouse embryonic stem cells, neuronal progenitor cells, and adult mouse brain tissue. This study identified Wdr76, Thy28, and Neil1 as general 5-hmC binding proteins and also discovered multiple cell-type specific 5-hmC binding proteins [25]. Despite this progress, these reports contain conflicting data about the affinity of different DNA binding proteins for modified cytosine species, and controversy exists to date as to which of these proteins and/or others are the true mediators of 5-hmC epigenetic function. We look forward to future work that will shed further light on this issue and determine the role of 5-hmC binding proteins in controlling gene expression.

## 2. Techniques Used to Study 5-hmC

### 2.1. The Limitations of Sodium Bisulfite Based Technologies

**Figure 2:**
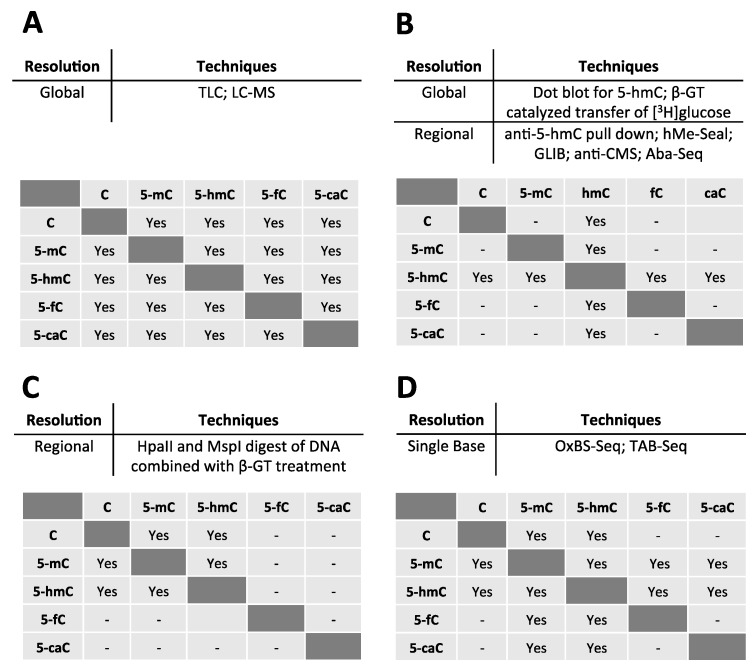
The capacity of various techniques to distinguish modified cytosine species from each other. The ability of a technique to distinguish cytosine species in the top row from those listed in the left column is indicated by a “Yes” entry. (**A**) TLC and HPLC-MS have the capacity to distinguish all modified cytosine species from each other at a global level of resolution. (**B**) At a global level of resolution, dot blots have the capacity to distinguish 5-hmC from all other cytosine species. Affinity purification of 5-hmC, hMe-Seal, GLIB, anti-CMS, and Aba-Seq have the capacity to distinguish 5-hmC from all other cytosine species at the regional level of resolution. (**C**) When combined with β-GT treatment of DNA, HpaII and MspI digestion of DNA has the capacity to distinguish cytosine, 5-mC, and 5-hmC. (**D**) When done in parallel with traditional bisulfite sequencing, oxidative bisulfite sequencing (OxBs) and Tet-assisted bisulfite sequencing (TAB-Seq) can distinguish various modified cytosine species at single base resolution.

Sodium bisulfite treatment of DNA followed by alkali desulphonation, hereafter referred to as sodium bisulfite treatment, has been the primary technology used to identify 5-mC at single-base or regional resolution. This treatment deaminates cytosine to uracil but does not deaminate 5-mC, allowing for cytosine and 5-mC to be distinguished. Technologies using sodium bisulfite treatment include: sodium bisulfite sequencing (e.g., reduced representation bisulfite sequencing, whole genome bisulfite sequencing, or site specific bisulfite sequencing), Illumina Infinium arrays, and methylation-specific PCR, among others. Unfortunately, the growing realization that 5-hmC is biologically important and distinct from 5-mC requires that data acquired from bisulfite-dependent technologies be re-interpreted, since 5-hmC is also protected from the bisulfite chemical reaction and therefore sequences as cytosine, like 5-mC [26,27]. Since sodium bisulfite cannot distinguish 5-hmC from 5-mC, numerous technologies that identify 5-hmC specifically in the genome at both the regional and single base resolution levels have been developed recently. Below, we describe these novel techniques in addition to techniques used to assay global levels of 5-hmC in the genome, some of which have been employed since the 1950s. Given the immense interest in 5-hmC, many techniques are now being used to study this base. As a result, this review will discuss some of the most commonly used techniques, but is not intended as an exhaustive discussion of all techniques in the field. The ability of various techniques to distinguish different cytosine species is summarized in Figure 2.

### 2.2. Detection of 5-hmC at the Global Level

Paper chromatography was used as early as the 1950s to distinguish 5-hmC from other bases [2,28,29]. More recently, a similar chromatographic method, thin layer chromatography (TLC), has been used to identify 5-hmC as well as 5-fC and 5-caC [6,7,8,9]. When performing TLC to detect modified cytosine species, DNA is typically digested by a methylation insensitive restriction enzyme and then labeled with γ-^32^P. DNA fragments are then digested to 5'dNMPs and resolved by TLC [6,7]. TLC separation of DNA bases is afforded by the fact that the bases have different polarities. As a result, each base interacts differently with the stationary and mobile phases used in the separation [30,31]. To identify 5-fC and 5-caC, two dimensional TLC is employed [8,9].

A second method used to measure global levels of 5-hmC is liquid chromatography-mass spectrometry (LC-MS) [6,7,32]. When running this assay, DNA is hydrolyzed to nucleosides by digestion with nuclease P1, phosphodiesterase, and alkaline phosphatase. The DNA bases are then separated by LC, ionized, and specifically detected and quantified by mass spectrometry [33]. LC-MS has the advantage of also being able to detect 5-fC and 5-caC [8,9].

A third method to detect global levels of 5-hmC is by transfer of radiolabeled glucose to 5-hmC. Glycosylation of 5-hmC is catalyzed by β-glucosyltransferase (β-GT), an enzyme encoded for by the T4 bacteriophage which contains high levels of glycosylated 5-hmC in its genome [34]. Thus, by incubating DNA with UDP-[^3^H]glucose and β-GT, 5-hmC residues are radiolabeled, allowing for easy and sensitive quantitation of global 5-hmC levels [35,36]. Importantly, this technique was the first to use β-GT as a method to label 5-hmC [35]. Labeling of 5-hmC by β-GT is the foundation for many of the techniques used to identify 5-hmC discussed in the following sections.

Finally, global levels of 5-hmC can be measured by dot blot. Dot blots can be performed with or without chemical labeling. When performed without chemical labeling, genomic DNA (gDNA) is denatured with NaOH and spotted on a membrane. An anti-5-hmC antibody is then used to probe for 5-hmC [37,38]. Performing dot blots without chemical labeling has the drawback of being dependent on anti-5-hmC antibodies, which can vary in sensitivity and specificity. Alternatively, dot blots can be performed after labeling DNA either with biotin through the hMe-Seal [39] or GLIB techniques [30], or by sulfonation through the anti-CMS technique [40]. These labeling strategies are outlined in the next section.

### 2.3. Detection of 5-hmC at the Regional Level

Multiple methods have been used to identify 5-hmC at the regional level in the genome. The simplest of these methods involves immunoprecipitation of DNA with an anti-5-hmC antibody followed by array-based detection or next generation sequencing of enriched DNA. Despite the simplicity of this approach, it is complicated by poor specificity of anti-5-hmC antibodies and being biased towards 5-hmC rich regions of the genome [21].

**Figure 3:**
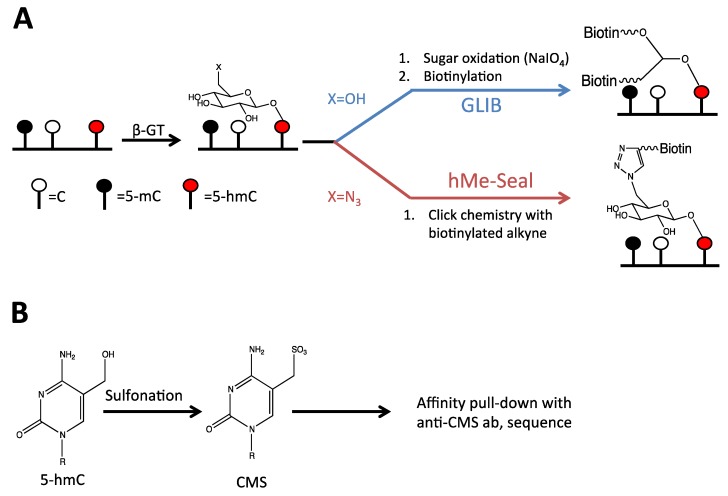
Techniques used to identify 5-hmC at a regional level of resolution. (**A**) In the GLIB and hME-Seal techniques for regional detection of 5-hmC, 5-hmC is first conjugated to a sugar using β-glucosyltransferase. In the GLIB technique, the conjugated sugar is glucose, whereas in the hMe-Seal technique, the glucose has been modified to carry an azide group. In the GLIB technique, the conjugated sugar is oxidized and biotinylated by nucleophilic addition of amines, whereas in the hMe-Seal technique the conjugated sugar is biotinylated through click chemistry. (**B**) Using the anti-CMS technique, 5-hmC is sulfonated with bisulfite and then pulled down using an anti-CMS antibody.

To circumvent these problems, various chemical labeling techniques have been developed. The first of these is referred to as hMe-Seal. In this technique, 5-hmC is conjugated to an azide-containing sugar by the catalytic activity of β-GT. Following conjugation to this glucose derivative, 5-hmC is labeled with biotin through click chemistry. DNA containing 5-hmC can then be selectively purified using avidin (Figure 3). As a result, this assay allows for extremely sensitive isolation of 5-hmC containing sequences with limited background [39].

A similar technique, GLIB (glycosylation, periodate oxidation, and biotinylation) also takes advantage of β-GT to label 5-hmC. Following conjugation of glucose to 5-hmC, glucose is oxidized by sodium periodate. The oxidation product possesses two aldehydes susceptible to nucleophilic attack by amine groups. By incubating the oxidized sugar with amines linked to biotin, 5-hmC is selectively biotinylated [30]. As in the hMe-Seal technique outlined above, this allows for selective isolation of 5-hmC containing DNA (Figure 3). A drawback to the GLIB technique is the high background generated by sodium periodate oxidation of DNA [21,30].

An anti-CMS (cytosine-5-methylene-sulfonate) antibody has also been developed to isolate regions of DNA containing 5-hmC. In this technique, DNA is treated with bisulfite causing 5-hmC to become sulfonated forming cytosine-5-methylene-sulfonate (CMS). 5-hmC-containing DNA can then be immunoprecipitated using an anti-CMS antibody (Figure 3) [40]. It is important to note that while deamination of cytosine in bisulfite treatment is facilitated by sulfonation of the pyrimidine ring, sulfonation of 5-hmC does not occur on the ring itself. 5-hmC is therefore not susceptible to deamination in the anti-CMS protocol. A potential drawback to the anti-CMS technique is that performing genome-wide sequencing experiments following bisulfite treatment is difficult, since cytosines have been converted to thymines. As a result, it is more difficult to map reads to the reference genome [41,42,43].

Finally, two approaches have been developed that use restriction enzymes to detect 5-hmC. In the first technique, MspI and HpaII, isoschizomers that cut at CCGG sites, are used to digest DNA. Since MspI is methylation insensitive but HpaII digestion is blocked by cytosine methylation, these enzymes have been used previously to identify cytosine methylation [44,45]. Kinney *et al.* have modified this procedure to include β-GT mediated glycosylation of DNA prior to digestion with these enzymes. Glycosylation blocks MspI digestion of hydroxymethylated DNA thereby allowing 5-mC and 5-hmC to be distinguished. A drawback to this approach is that only CpGs in the CCGG context are probed for hydroxymethylation [46].

The second restriction enzyme based approach uses the AbaSI enzyme, which cuts DNA near glycosylated cytosines. Most frequently, the enzyme cuts 11-13 nucleotides from a glycosylated 5-hmC in the top strand, and 9-11 nucleotides away in the bottom strand [47,48]. In the Aba-Seq protocol, genomic DNA is glycosylated with β-GT and then digested with AbaSI. After adapter ligation, sequencing can identify AbaSI cut sites. Since AbaSI prefers cutting DNA when there are cytosines arranged symmetrically around the cleavage site, the technique makes assumptions about the likely location of 5-hmC in order to determine which cytosine around the cut site should be mapped as 5-hmC (e.g., the authors assume that 5-hmC is more likely to be at a CpG cytosine than a non-CpG cytosine). Using these assumptions, the authors are able to determine the location of 5-hmC with high confidence for 82% of cleavage sites. This technique has the advantage of being able to identify 5-hmC sites with low sequencing depth and using small quantities (as low as 50 ng of mouse E14 genomic DNA) of DNA [48].

### 2.4. Detection of 5-hmC at the Single Base Level

**Figure 4:**
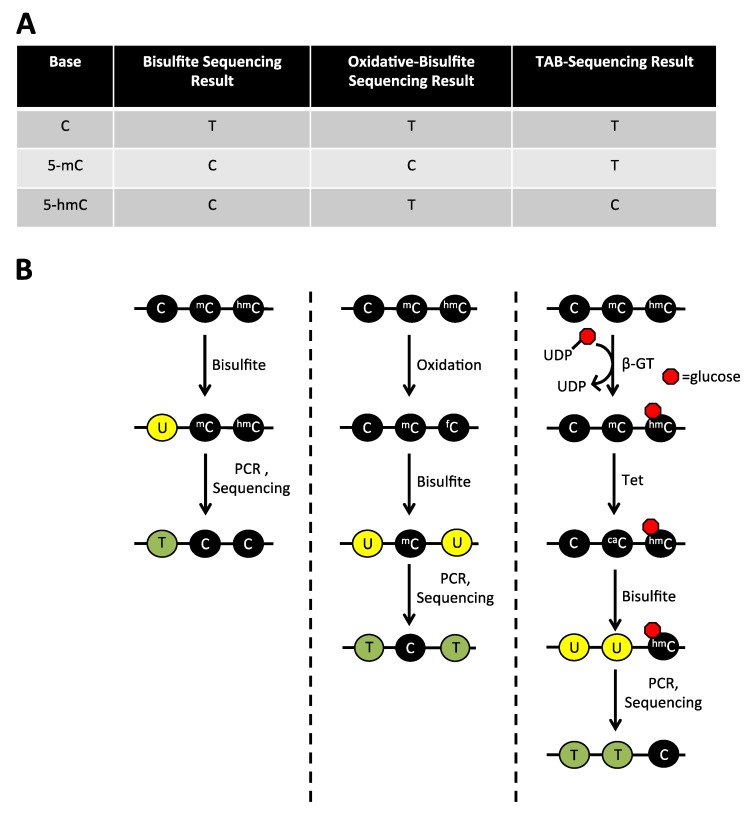
Techniques used to distinguish modified cytosine species at single base resolution. (**A**) In traditional bisulfite sequencing, bisulfite converts cytosine to uracil (yellow circles), which is then sequenced as a thymine (green circles), whereas 5-mC and 5-hmC remain unchanged. (**B**) In oxidative bisulfite sequencing, an additional oxidative step is included before bisulfite treatment that converts 5-hmC to 5-fC. 5-fC is then converted to uracil on bisulfite treatment so that both cytosine and 5-hmC are read as thymines during sequencing of oxidative-bisulfite treated DNA. (**C**) Finally, in Tet-assisted bisulfite sequencing (TAB-Seq), all 5-hmC residues are conjugated to glucose (small red circle). This protects them from oxidation during treatment with Tet, which converts all modified cytosines, except 5-hmC, to 5-caC. On bisulfite treatment, only glucose conjugated 5-hmC residues are protected from deamination and are read as cytosines during sequencing.

Three techniques have been developed to identify 5-hmC at single base resolution within the genome. The first technique, referred to as oxidative bisulfite sequencing, adds an oxidative step before traditional bisulfite treatment of gDNA. In this step, DNA is treated with potassium perruthenate (KRuO_4_), which oxidizes 5-hmC into 5-fC. On treatment with bisulfite, 5-fC (like cytosine) is converted to uracil, which is then PCR amplified and sequenced as thymine. By performing oxidative bisulfite sequencing in parallel with traditional bisulfite sequencing, the location of 5-hmC can be inferred by finding residues converted to thymine in oxidative-bisulfite sequencing, but not in traditional bisulfite sequencing (Figure 4) [49].

The second method is Tet-assisted bisulfite sequencing (TAB-Seq). In this method, gDNA is treated with β-GT to conjugate all 5-hmC residues to glucose. The DNA is then treated with Tet1 to convert 5-mC and 5-fC to 5-caC, while cytosine and glycosylated 5-hmC remain unaffected. During subsequent bisulfite treatment, unmodified cytosines and 5-caC are converted to uracil or 5-carboxyuracil (5-caU), respectively, whereas 5-hmC remains protected by glycosylation. The location of 5-hmC is then indicated by a cytosine in sequencing results since all other cytosine species (C, 5-mC, 5-fC, and 5-caC) have been converted to thymine (Figure 4) [50]. This technique has the advantage of identifying 5-hmC directly, without comparing results to traditional bisulfite sequencing.

Despite this progress, oxidative-bisulfite sequencing and TAB-Seq suffer the same drawback as the anti-CMS technique when applied to the genome-wide level: Mapping reads from next generation sequencing data to reference genomes becomes difficult after bisulfite treatment since the sequence of bisulfite treated DNA no longer matches that of the reference genome [41,42,43].

A final technique capable of identifying 5-hmC at single base resolution uses single-molecule, real-time sequencing (SMRT). This technology detects modified nucleotides in a DNA sequence as changes in polymerase kinetics during DNA synthesis. By itself, this technique can detect 5-mC [51]. When combined with β-GT labeling and affinity purification of 5-hmC containing DNA by the hMe-Seal technique, SMRT can be used to detect 5-hmC [52].

## 3. The Role of Hydroxymethylation in Normal Physiology

We will review the role of 5-hmC briefly in normal physiology, since its role in normal cellular processes sheds light on how alterations to 5-hmC regulation may aid tumorigenesis. For example, evidence from studying fertilization, discussed below, shows how Tet activity can be used to activate gene transcription. Thus, interfering with Tet activity could be a mechanism used by cancer cells to repress gene transcription.

### 3.1. Demethylation of the Paternal Genome after Fertilization

More than a decade ago, it was observed that the paternal genome undergoes rapid demethylation after fertilization of the oocyte [53,54]. The mechanism behind this process has recently become understood. After fertilization, loss of 5-mC in the paternal pronucleus coincides with gain of 5-hmC [55,56,57,58]. Among the *Tet* genes, *Tet3* has the highest expression in the zygote, and Tet3 localizes selectively to the paternal pronucleus [56,59]. Tet3 depletion inhibits paternal genome demethylation, leads to incomplete activation of paternal copies of genes such as *Oct4*, and reduces fetal survival, confirming its function in paternal genome demethylation [58]. Although Tet3 in the zygote is maternally derived, the maternal genome is protected from Tet3-mediated hydroxymethylation by PGC7 which selectively localizes to the maternal pronucleus, because it is rich in histone 3 lysine nine dimethylation (H3K9me2) [55,60].

### 3.2. Embryonic Stem Cells (ESCs)

Studies from ESCs suggest that Tet activity and 5-hmC are involved in regulating the balance between pluripotency and differentiation, which is often disrupted in cancers. In mouse ESCs, *Tet1* is expressed at highest levels, *Tet2* at intermediate levels, and *Tet3* at the lowest levels [6,61]. *Tet1* may be important in maintaining transcription of pluripotency genes [62], including *Nanog* [63]*.* However, regulation of *Nanog* by Tet1 was not observed by others [61]. Physical interaction of Tet1 and Tet2 with Nanog have also been reported as a mechanism to regulate pluripotency genes [64]. Tet1 is important in determining lineage commitment *in vitro* since its depletion skews ESC differentiation towards trophoectoderm [61,62,63], but the importance of embryonic *Tet1* and hydroxymethylation *in vivo* is unclear, since *Tet1* knockout mice develop normally with no overt abnormalities except a slightly smaller body size at birth [65]. This may be due to redundancy between Tet1 and Tet2 functions in ESCs, which are both expressed at appreciable levels. Therefore, *Tet1-Tet2* double knockout mice were generated. Many of these mice die during gestation or perinatally. Nonetheless, some double knockout mice survive to adulthood, but this may be due to increased embryonic expression of *Tet3* in *Tet1-Tet2* double knockout mice [66]. As a result, the *in vivo* embryonic importance of Tet1 and Tet2 in ESCs remains unclear.

ESCs are unique in that Tet1 has also been associated with gene repression [67], including through recruitment of the polycomb repressor complex [68]. Although Tet-mediated gene repression via the polycomb complex has not yet been studied in cancer, this observation suggests that interfering with Tet activity has the potential to have bidirectional effects on tumor cell gene expression.

### 3.3. Hydroxymethylcytosine in the Brain

5-hmC levels are highest in the brain compared to other differentiated tissues, suggesting that this base plays an important role in nervous system physiology [69]. Moreover, since altered 5-mC and 5-hmC levels are detected in gliomas, changes in the pathways regulating 5-hmC in the brain likely play a role in glioma tumorigenesis, a topic discussed further in detail below.

In the cerebellum, 5-hmC is most enriched within gene bodies, and the extent of hydroxymethylation positively correlates with gene expression [39,70]. This correlation, however, might be cell-type specific, since Mellen *et al*. found that 5-hmC correlated with expression in granule and Bergmann cells, but not in Purkinje cells [24]. Nonetheless, even in Purkinje cells, the 5-hmC/5-mC ratio was a better predictor of gene expression than methylation alone [24].

Although Khare *et al*. found a positive correlation between gene hydroxymethylation and expression in multiple tissues, this did not reach statistical significance in the brain. This may be because hydroxymethylation conveys additional biological function beyond controlling gene expression in the brain. Specifically, they found that 5-hmC levels change over exon-intron boundaries, and that hydroxymethylation levels were lower at alternatively spliced exons, suggesting that 5-hmC may direct the splicing machinery [71].

## 4. Glioma

### 4.1. 5-hmC Levels Are Reduced in Gliomas

Gliomas are cancers of the central nervous system that arise from the glial cells of the brain. Along with other clinical findings, including age of the patient, neurologic performance status, and tumor location, the WHO classification of glioma tumor grade predicts patient response to therapy and outcome [72]. Whereas patients with grade II gliomas typically survive more than 5 years, patients with grade IV gliomas, also known as glioblastoma multiforme, have a median survival of only 15 months [73]. Two studies have investigated the disruption of normal cytosine modification patterns in human gliomas [74,75]. The discovery of 5-hmC, and the fact that it is present at high levels in the brain, led several research groups to investigate the extent to which 5-hmC patterns were disrupted in gliomas. Orr and colleagues observed that 5-hmC levels decreased by grade, with low-grade tumors showing high levels of 5-hmC, and glioblastomas showing the lowest levels of 5-hmC. Accordingly, low levels of 5-hmC in adult glioblastoma and grade II or III astrocytomas correlated with poor prognosis [76] Kraus and colleagues also observed high numbers of 5-hmC positive cells in WHO grade I gliomas, fewer in grades II and III, and the least number of 5-hmC positive cells in grade IV gliomas [77].

### 4.2. Potential Mechanisms for Loss of 5-hmC in Gliomas

As discussed above, the TET enzymes convert 5-mC to 5-hmC, whereas the IDH enzymes provide the cofactor α-ketoglutarate needed for the TET enzymes to perform catalysis. Disruption of either enzyme family may be responsible for loss of 5-hmC in glioma. *IDH1/2* mutations are common in several cancers, including gliomas [78]. *IDH1/2* mutations produce the oncometabolite 2-hydroxyglutarate (2-HG), which acts as a competitive inhibitor of α-ketoglutarate-dependent dioxygenases, including the TET enzymes [37]. The TET enzymes may also be disrupted through multiple mechanisms leading to reduced levels of 5-hmC in gliomas. Finally, loss of 5-hmC may be due to increased activity of the deaminases and base-excision repair (BER) enzymes, which are involved in substituting 5-hmC for unmodified cytosine [13].

### 4.3. IDH1/2 Mutations

A significant proportion of low grade gliomas (>85%) and a smaller proportion of glioblastomas contain mutations in *IDH1* or *IDH2*, which have been shown to associate with a specific hypermethylated phenotype and predict better overall survival for those patients [79,80,81,82,83]. Mutations in *IDH1/2* are thought to occur early in the progression of gliomas, and some studies of other human cancers, such as leukemia (see below), have suggested a link between *IDH* mutations and loss of 5-hmC [84]. As a result, several groups searched for associations between *IDH* mutations and loss of 5-hmC in gliomas [85,86]. Liu *et al.* detected slightly lower 5-hmC levels in *IDH* mutant astrocytomas [86], and Turcan *et al.* demonstrated that expression of mutant IDH in astrocytes reduces 5-hmC levels [83]. Nonetheless, other mechanisms probably also lead to 5-hmC loss in glioma. In fact, Müller and colleagues found that 68% of gliomas with wild-type *IDH* do not express detectable levels of 5-hmC [85].

### 4.4. TET Silencing or Mislocalization

Another potential mechanism for reduced 5-hmC levels is disruption of TET enzyme expression or activity. Kim and colleagues examined low-grade gliomas with wild-type *IDH1/2* for mutations in or hypermethylation of *TET2* [87]. Although they found no mutations in *TET2*, they identified promoter methylation of *TET2* in 5 of 35 low-grade gliomas, whereas low-grade gliomas with *IDH1/2* mutations showed no hypermethylation of the *TET2* promoter. Considering loss of 5-hmC is most prominent in high-grade gliomas, it is possible that silencing of *TET2* by DNA methylation would have been found more frequently in higher grade gliomas. Another possibility is that either TET1 or TET3 drives conversion of 5-mC to 5-hmC in the brain and that their activity is disrupted in glioma.

Müller and colleagues investigated levels of 5-hmC and the expression of TET1 and TET2 in glioma tissues and cell lines [85]. Real-time RT-PCR analysis of 54 glioma samples showed that *TET1* and *TET2* mRNA was expressed at varying levels in primary glioblastomas, secondary glioblastomas, and anaplastic astrocytomas. However, they found that tumors lacking detectable levels of 5-hmC often showed no expression of *TET1* or mislocalization of TET1 to the cytoplasm. In addition, all six glioblastoma cell lines examined showed nuclear exclusion of TET1, whereas TET2 was detected in the nuclei of all glioma samples and cell lines. Thus, the authors suggest that TET1 nuclear exclusion contributes to the loss of 5-hmC in these tumors [85].

### 4.5. Removal of 5-hmC by Base Excision Repair Enzymes

Homeostasis of 5-hmC levels is regulated in part by deaminases (AID/APOBEC) and the BER pathway, which can ultimately replace 5-hmC with unmodified cytosine. Using the publicly available TCGA glioblastoma dataset, Orr and colleagues demonstrated that expression of 6 of 10 AID/APOBEC genes and 2 of 5 BER genes were increased in the mesenchymal subtype of glioblastoma compared to the proneural subtype [76]. These subtypes were defined by TCGA based on the tumor’s genomic characteristics and the patient’s age at diagnosis, response to treatment, and survival time. In keeping with the fact that patients with tumors of the proneural subtype are younger and tend to survive longer compared with the other subtypes [88,89], Orr and colleagues detected that high expression of *APOBEC3G* correlated with reduced survival, which they validated in REMBRANDT, an independent dataset [76].

Although the molecular mechanism behind the loss of 5-hmC in gliomas is not yet completely understood, these studies show an obvious disruption of several enzymes needed to form and remove this epigenetic mark. Elucidating the different mechanisms at play in individual patients may allow for a more personalized, targeted therapeutic strategy with the development of therapies that modulate these pathways.

## 5. Hematological Malignancies

As in the brain, disruption of the balance of covalent cytosine modifications is found in many types of hematopoietic malignancies. Acute myeloid leukemia (AML) with a normal karyotype (CN-AML), which accounts for 40–50% of AML cases, commonly presents with genetic mutations in *ASXL1*, *MLL*, *DNMT3A*, *TET2*, *IDH1* and *IDH2* genes*,* all of which have important roles in epigenetic regulation. Mutations in *TET2* or *IDH1/IDH2* disrupt 5-hmC homeostasis, resulting in inadequate maintenance of 5-hmC in hematopoietic progenitor and stem cells [90,91,92].

### 5.1. TET Enzymes in Hematological Malignancies

**Figure 5:**
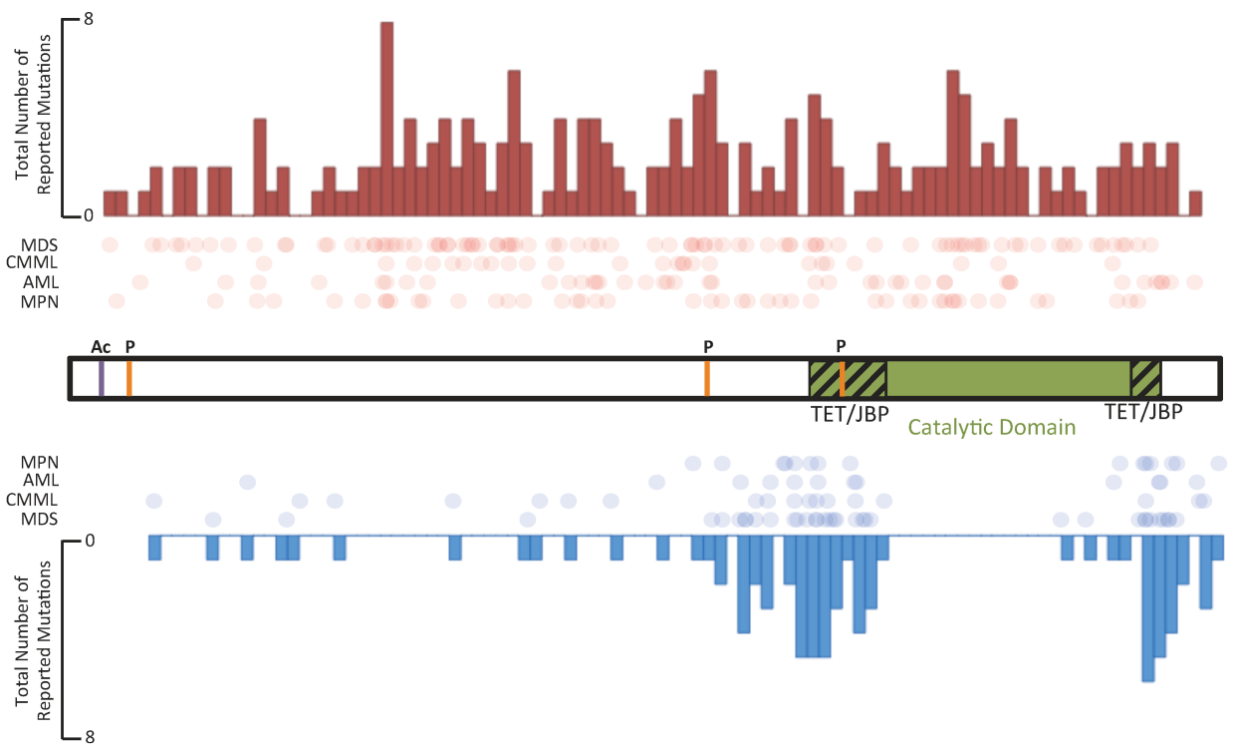
The overall frequency of nonsense and frameshift mutations and missense mutations in TET2. The TET2 protein is shown schematically in the center of the figure. Phosphorylation and acetylation sites are marked on the protein schematic in orange and purple, respectively. The catalytic domain, which is composed of two TET/JBP domains separated by a spacer region, is highlighted in green near the C-terminus. The primary protein structure was divided into seventy-five bins, and the total number of mutations reported for each bin [84,93,95,101,114] is indicated in the histograms. At the top of the figure, nonsense and frameshift mutations are shown as red circles; and at the bottom of the figure, missense mutations are shown as blue circles.

As discussed above, *TET1* was first identified as a fusion partner of the mixed lineage leukemia (*MLL*) gene in adult and pediatric leukemias with the translocation t(10:11)(q22;q23) [4,5]. Six years later, *TET2* somatic mutations were identified in myeloproliferative neoplasms (MPN) and myelo-dysplastic syndrome (MDS) [93,94]. Among hematological malignancies, *TET2* is mutated most frequently in AML, secondary AML (sAML), myelodysplastic syndrome (MDS), systemic mastocytosis, chronic myelomonocytic leukemia (CMML), and other MPNs [94,95,96,97,98,99,100,101,102,103,104,105,106]. Although frameshift and nonsense mutations of *TET2* occur throughout the entire gene, point mutations are found within exons encoding the TET/JBP component of the catalytic domain (Figure 5). Loss of TET2 catalytic activity is correlated with low genomic 5-hmC levels [38]. The observation of heterozygous *TET2* mutations suggests that *TET2* is haploinsufficient or the mutations have dominant-negative effects [107].

Although a global decrease in 5-hmC levels might suggest there would be a corresponding increase in global levels of 5-mC, the reported effects of TET2 mutations on 5-mC level in patients have been unclear. Ko *et al*. reported global hypomethylation [38,84], whereas Figueroa *et al*. demonstrated global hypermethylation [38,84]. These differences may be the result of these groups using different techniques (the Illumina Infinium 27K methylation array versus HPLC-MS and the HELP assay, respectively), or because they studied different diseases (MDS/MPN, primary and secondary AML versus AML, respectively) [38,84]. In agreement with the results from Figueroa *et al*., samples from CMML patients with *TET2* mutations have also demonstrated significant global hypermethylation [107].

Several *Tet2* knockout mouse models have been developed. These mice display increased stem cell self-renewal and hematopoietic transformation *in vivo*. One of these models, published by Moran-Crusio, carried a conditional knockout of the *Tet2* allele and showed progressive hematopoietic stem cell (HSC) expansion and myeloproliferation (neutrophilia, monocytosis, and splenomegaly). Importantly, this model also confirmed that *Tet2* haploinsufficiency is sufficient to promote HSC self-renewal and myeloproliferation *in vivo* [91]. In a parallel publication, Quivoron *et al*. showed with two mouse models, a gene-trap and a conditional knockout, that alteration of Tet2 function resulted in pleiotropic hematopoietic abnormalities. The gene-trapped *Tet2* knockout mouse developed myeloid malignancies that were transplantable to secondary recipients, while the conditional knockout mouse did not die from hematological disease. They also observed that *Tet2* inactivation altered T and B cell differentiation [108]. Other *Tet2* knockout mouse models have also been characterized as having a similar, CMML-like phenotype of increased HSC and myeloid proliferation that corresponds to CMML [61,90,109,110]. Moreover, depletion of *TET2* by RNA interference in cord blood CD34^+^ cells skews progenitor differentiation toward the granulo-monocytic lineage at the expense of lymphoid and erythroid lineages [111]. Taken together, these mouse studies suggest that loss of *Tet2* promotes myelomonocytic expansion and illustrates that the TET2 enzyme plays a significant role in stem cell development and differentiation.

Acquisition of *TET2* mutations is an early clonal event in MPN with mutations in Janus kinase 2 (*JAK2*) and myeloproliferative leukemia oncogene (*MPL*), implying that loss of 5-hmC levels may have a functional role in the onset and/or progression of hematological malignancies [96,112].

There is no definitive conclusion about the prognostic significance of *TET2* mutations. To date, results from published studies have not demonstrated any significance of *TET2* mutations in the outcome for patients with MPN or MDS. However, they have demonstrated a worse outcome for CN-AML patients that otherwise have favorable prognoses [113,114]. In a cohort of Chinese patients with MDS, low 5-hmC levels, but not necessarily *TET2* mutations, correlated with poor prognosis [115].

### 5.2. IDH Enzymes in Hematological Malignancies

Mutations in *IDH1* were first identified in colorectal adenocarcinomas [116]. Subsequently, mutations in both *IDH1* and *IDH2* were found in more than 70% of gliomas and secondary glioblastomas (discussed above) [81,117], as well as in AML, with a frequency of approximately 15% [118].

IDH1/2 are key functional enzymes in the Krebs cycle, where they catalyze the conversion of isocitrate to α-ketoglutarate (α-KG) while reducing NAD^+^ to NADH. IDH1 is localized in the cytoplasm or in peroxisomes, whereas IDH2 is localized in mitochondria. Mutations in *IDH1* and *IDH2* lead to an aberrant gain-of-function phenotype that enables these enzymes to convert isocitrate to 2-hydroxyglutarate (2-HG) instead of its normal product, α-KG. Subsequently, 2-HG accumulates at high levels in cells and tissues [119,120], where it acts as a oncometabolite by interfering with the normal function of enzymes that use α-KG as their normal substrate (Figure 1), like the TETs and jumonji-domain-containing (JMJC) family of histone lysine demethylases that act on histone H3 [37,86,121,122].

It has recently been shown that the (R)-enantiomer of 2-hydroxyglutarate ((R)-2-HG) is sufficient for leukemic cell transformation, since human erythroleukemia cells became cytokine independent upon transformation with an IDH1-R132H mutant construct that produced (R)-2-HG [123]. Interestingly, the (S)-enantiomer of 2-HG is an even more potent inhibitor of *TET2*, but does not possess this transforming ability. This paradox can be explained by the involvement of Egl nine homolog 1 (EGLN1), also known as prolyl hydroxylase domain-containing protein 2 (PHD2), in the transformation event. (R)-2-HG acts as an agonist of EGLN1, whereas (S)-2-HG is antagonist of EGLN1. Loss of EGLN1 activity blocks transformation driven by mutant IDH or TET2, accounting for the inability of (S)-2-HG to transform these cells [124]. Because the level of 2-HG is highly elevated in *IDH1/2*-mutated AML, measuring 2-HG levels in patient samples could be used potentially for diagnosis and monitoring of disease progression. This is supported by work showing that in *IDH* mutant AML, 2-HG levels decrease and increase with disease remission and relapse, respectively [125]. Furthermore, competitive inhibitors with ∼90% efficacy for the IDH1-R132H mutation have recently been synthesized [126], creating the possibility for targeted therapies of AMLs with *IDH1/2* mutations.

The reduced frequency of recurrent chromosomal aberrations and other AML-associated mutations in *IDH1*/*2*-mutant leukemias implies that mutations in *IDH1/2* may represent a distinct mechanism for AML pathogenesis. A common feature of CN-AML with *IDH1/2* mutations is a 10 to 100-times higher level of 2-HG compared with IDH wild-type AMLs. This is consistent with the gain-of-function phenotype of the mutant enzyme [127]. Data collected as a part of the ECOG E1900 clinical trial, which includes 385 primary leukemia samples, demonstrates that 2-HG reduces 5-hmC levels and promotes global DNA hypermethylation [84]. The role of 2-HG in inhibiting TET-mediated conversion of 5-mC to 5-hmC is also supported by the finding of an inverse correlation between 2-HG levels and 5-hmC levels, and a positive correlation between 2-HG levels and 5-mC levels in patients with *IDH* mutant AML [125]. These findings in hematological malignancies are in agreement with the hypermethylator phenotype that has been described in *IDH* mutant gliomas [83].

Mutations of the *IDH1* and *IDH2* genes have certain prognostic value. Although the IDH1-R132H and IDH2-R172K mutations have not been demonstrated to influence prognosis, the IDH2-R140Q mutation is associated with a favorable clinical outcome, as shown in a large dataset with over 1000 patients enrolled in the UK MRC AML 10 and AML 12 clinical trials [128,129]. Moreover, *IDH1/2* mutations often co-occur with mutations in *NPM1*, which is, in itself, favorable [130]. Concurrent *IDH1* and *IDH2* mutations in AML are absent [131] or very rare [118,132,133], and *TET2* and *IDH* mutations are mutually exclusive [84]. In primary myelofibrosis, *IDH* mutations may identify patients at risk for premature death and/or leukemic transformation [134].

## 6. Hydroxymethylation in Other Solid Tumors

Alterations to 5-hmC have been less studied in solid tumors other than glioma. The first study that characterized 5-hmC in human solid tumors was by Haffner *et al*. in which the authors quantified 5-hmC levels in formalin-fixed paraffin-embedded human cancer tissues by immunohistochemistry [135]. This method allowed them to measure hydroxymethylation on a cell-by-cell basis. Carcinomas of the prostate, breast, and colon showed a significant reduction of 5-hmC levels compared to corresponding normal tissues. Compared with 5-hmC loss, only moderate reductions of 5-mC were observed in prostate and colon cancer, which suggests that 5-hmC loss in these carcinomas is not simply the result of decreased 5-mC [135].

Other groups have since provided further evidence for the reduction of 5-hmC in solid tumors. Seung-Gi Jin *et al*. found a 5-fold decrease of 5-hmC by LC-MS/MS in stage I squamous cell carcinomas of the lung with respect to matched normal tissue samples [136]. In some of these cases, 5-mC was also significantly reduced suggesting that sometimes 5-hmC loss is secondary to 5-mC reductions [136]. Future work could determine this by investigating whether regional 5-hmC losses overlap with regional 5-mC losses in these cases using techniques capable of distinguishing 5-mC and 5-hmC with gene-level resolution. Additionally, IHC results demonstrated a decrease in 5-hmC levels in invasive ductal carcinoma of the breast, hepatocellular carcinoma, renal cell carcinoma, squamous cell carcinoma of the lung, rhabdomyosarcoma, melanoma, malignant mesenchymoma of the small intestine, and adenocarcinomas of the pancreas, prostate, stomach, uterus, and ovary [136]. The finding of decreased 5-hmC levels in breast [137] and hepatocellular carcinomas [138] has also been recapitulated in two recent publications.

The mechanisms of 5-hmC reduction in solid tumors are far from being understood fully. However, just like in gliomas and hematological malignancies, TET and IDH proteins are thought to play an important role. In breast and liver cancers, expression of all three *TET* genes has been shown to decrease along with 5-hmC reduction [137], and in colon cancer *TET1* down-regulation has been reported [139]. In melanoma, down-regulation of *TET*, especially *TET2*, and *IDH2* transcripts have been reported as mechanisms of 5-hmC loss [140,141]. Although *IDH1* mutations have been identified in 10% of melanomas in one patient cohort [142], *TET* and/or *IDH* gene repression seems to be a more common mechanism for blocking 5-mC to 5-hmC conversion in non-glioma solid tumors rather than *TET* or *IDH* mutations, which are found more commonly in gliomas and hematological malignancies.

The downstream effects of altered TET activity are unclear. *TET1* down-regulation is not sufficient to transform 3T3 cells or increase their colony forming capacity [139], but altered *TET* expression and 5-hmC levels may be either necessary or facilitative for transformation. This is supported by the fact that restoring *TET2* expression slows melanoma cell growth in mice xenografts and overexpression of *IDH2* in a zebrafish model of melanoma improves tumor-free survival [140]. Few studies on solid tumors have had the capacity to determine the genes at which 5-hmC is being lost in solid tumors. By performing KEGG pathway analyses, Lian *et al*. found that loss of 5-hmC and gain of 5-mC in melanoma occurs over gene bodies of genes in the adherens junction pathways, Wnt signaling pathways, pathways in cancer, and melanogenesis pathways [140]. However, this study did not examine how changes in 5-hmC affected gene transcription. Hsu *et al*. found that TET1 normally maintains expression of tissue inhibitors of metalloproteinases (TIMPS) and that down-regulation of *TET1* in breast cancer facilitates migration and invasion of cancer cells and correlates with advanced tumor stage and poor survival [143]. Beyond this study, the transcriptional effects that result from 5-hmC loss in solid tumors remain an open question.

## 7. Common Themes/Conclusions

Studies addressing the role of 5-hmC in cancer have revealed loss of 5-hmC to be associated commonly with tumorigenesis in both hematological diseases and solid tumors. Whether this decrease in 5-hmC is accompanied by an increase in global 5-mC levels might be tumor type dependent. The mechanism by which 5-hmC loss induces tumor progression, or even initiation, will only be determined by identifying the genes and signaling pathways that are regulated by changes in 5-hmC in tumor evolution. Moreover, to gain a full understanding of how gene specific changes in 5-hmC facilitate tumor evolution, it will be critical to determine how changes in 5-hmC levels alter transcription. Evidence suggests that 5-hmC, especially over gene bodies, is associated with high transcriptional activity, and therefore, it is likely that global 5-hmC loss is used to transcriptionally inactivate certain tumor suppressors.

Even though 5-hmC loss is common in cancers studied to date, the mechanism by which 5-hmC levels are reduced varies. Hydroxymethylation is decreased in some hematological diseases and gliomas when the genes encoding for TET and IDH enzymes are mutated, whereas in solid tumors the same end result is achieved by down-regulation of *TET* and *IDH* transcription. The reasons for these different mechanisms remain unclear.

Finally, to increase our understanding of 5-hmC in both normal and cancerous cells, it is important to gain an understanding of how the 5-hmC mark is interpreted by the cell. This requires working towards a more complete knowledge of how 5-hmC binding proteins interact with other proteins to affect transcription.
